# Predictive value of *TP53* RNAscope^®^*in situ* hybridization and p53 immunohistochemistry for *TP53* mutational status in canine diffuse large B-cell lymphoma

**DOI:** 10.1080/01652176.2024.2403453

**Published:** 2024-09-16

**Authors:** Greta Foiani, Luca Licenziato, Laura Marconato, Antonella Fanelli, Erica Melchiotti, Claudia Zanardello, Luca Aresu, Marta Vascellari

**Affiliations:** a Histopathology Laboratory, Istituto Zooprofilattico Sperimentale delle Venezie, Padua, Italy; b Department of Veterinary Sciences, University of Turin, Grugliasco, Italy; c Department of Veterinary Medical Sciences, University of Bologna, Bologna, Italy

**Keywords:** Canine diffuse large B-cell lymphoma, immunohistochemistry, *in situ* hybridization, mutation, p53, *TP53*

## Abstract

*TP53* mutations are associated with short survival and poor treatment response in canine diffuse large B-cell lymphoma (cDLBCL). The expression of *TP53* by RNAscope^®^ *in situ* hybridization and p53 by immunohistochemistry (IHC) was investigated in 37 formalin-fixed paraffin-embedded cDLBCL, to assess their correlation with *TP53* mutational status and to evaluate their prognostic value. *TP53* was detected in all samples by RNAscope^®^. Ten of 37 (27%) cases expressed p53 by IHC, with highly variable percentage of positive cells. *TP53* RNAscope^®^ scores and p53 IHC results were not correlated. The expression of *TP53* by RNAscope^®^ was not influenced by its mutational status. Conversely, p53 IHC and *TP53* mutations were significantly associated. p53 IHC predicted *TP53* genetic mutations with high accuracy (97.3%). All *TP53-*mutated samples carrying missense mutations exhibited p53 expression by IHC, while all wild-type cases and a single case with frameshift insertion were negative. In univariable analysis, p53 IHC was associated with shorter time to progression (TTP) and lymphoma-specific survival (LSS). Nevertheless, in multivariable analysis, only treatment significantly affected TTP and LSS. These findings suggest p53 IHC is an accurate, cost-effective tool for predicting *TP53* mutations in cDLBCL, unlike *TP53* RNAscope^®^, though its prognostic value requires further validation.

## Introduction

Lymphoma is the second most prevalent cancer in dogs, following mammary tumors, with an estimated incidence rate ranging from 20 to 100 cases per 100,000 dogs (Avery 2020). Among lymphoid neoplasms, diffuse large B-cell lymphoma (DLBCL) is the most common, representing approximately half of all lymphomas in this species (Aresu 2016; Avery 2020). Canine DLBCL (cDLBCL) is characterized by aggressive behavior, short survival times, and a high relapse rate (Aresu 2016). The gold-standard therapeutic approach is chemotherapy, notably CHOP-based protocols (Childress et al. 2018). However, the recent integration of immunotherapy, specifically the administration of autologous vaccines like APAVAC^®^, has led to a significant improvement in outcome (Marconato et al. 2019). Nevertheless, the heterogeneous response to these therapeutic modalities underscores the molecular complexity of this tumor (Marconato et al. 2019).

Recent studies employing high-throughput technologies have allowed for an in-depth exploration of the molecular, epigenetic, and genetic features of cDLBCL, highlighting recurrent mutations in several genes, including *TRAF3*, *SETD2*, *POT1*, and *TP53* (Aresu et al. 2019; Giannuzzi et al. 2022; Fanelli et al. 2022). *TP53* aberrations, primarily consisting of missense mutations affecting the DNA-binding domain, have been identified in approximately 25% of cDLBCL (Giannuzzi et al. 2022). Notably, *TP53* mutations have emerged as key factors associated with shortened survival times in affected dogs, regardless of the therapeutic regimen (Giannuzzi et al. 2022). Indeed, the development of a recent predictive model incorporating *TP53* mutational status has identified a subgroup of dogs exhibiting limited benefit from the addition of immunotherapy to standard CHOP-based chemotherapy (Giannuzzi et al. 2022).

*TP53*, a key tumor suppressor gene encoding p53 protein, stands as the most commonly mutated gene in human cancers (Zhang et al. 2020). p53 orchestrates crucial biological processes such as cell cycle arrest, apoptosis, senescence, DNA repair, cellular metabolism, and antioxidant defense (Levine 2019). Mutations of the *TP53* gene can lead to the loss of normal p53 tumor suppressor functions (loss-of-function, LOF), the acquisition of oncogenic activities (gain-of-function, GOF), and dominant-negative effects, wherein mutant p53 inhibits wild-type p53 function (Miller et al. 2016; Attardi and Boutelle 2024).

Similar to cDLBCL, the majority *of TP53* mutations in human cancers are missense mutations, resulting in the production of full-length mutant p53 proteins (Zhang et al. 2020). While wild-type p53 protein undergoes rapid degradation, mutant p53 is often stabilized, leading to its accumulation in tumor cells (Xu et al. 2014; Zhou et al. 2022). Due to this characteristic, immunohistochemistry (IHC) has been widely employed as a surrogate for detecting *TP53* mutation in several human cancers (Bártek et al. 1991). Nevertheless, interpreting IHC results may be challenging due to the non-uniform frequency of *TP53* mutations across tumors, and immunohistochemical staining may be influenced by various analytical and pre-analytical conditions (Köbel et al. 2019). Similar to IHC, *in situ* hybridization (ISH), which targets nucleic acid sequences, serves as a highly sensitive biomarker assay that preserves cancer spatial information (Wang et al. 2012).

In the canine species, p53 immunoreactivity has been previously assessed for various tumors, using anti-human antibodies that recognize both wild-type and mutated p53 and are cross-reactive with canine tissues (Albaric et al. 2001; Keller et al. 2007; Brunetti et al. 2023). Few studies have investigated p53 immunohistochemical expression in canine lymphomas, including cDLBCL, without correlating the results with the *TP53* mutational status (Gamblin et al. 1997; Sueiro et al. 2004; Sokołowska et al. 2005; Dhaliwal et al. 2013).

In cDLBCL, the mechanisms underlying the role of *TP53* mutation in tumorigenesis are not fully understood and no studies have been conducted to correlate the mutational status, mRNA, and protein expression of *TP53*. This study aimed to assess the correlation of *TP53* RNAscope^®^ ISH and p53 IHC in formalin-fixed and paraffin-embedded (FFPE) cDLBCL samples with *TP53* mutational status and to explore their clinical and prognostic significance.

## Materials and methods

### Selection of dogs

Client-owned dogs with cDLBCL of any WHO stage were included in the analysis. Some of the dogs included here have been described previously in another study (Marconato et al. 2019). Specifically, dogs for which material was still available to conduct the analyses required for this research were included.

The study did not fall within the application areas of the Italian Legislative Decree 26/2014 which governs the protection of animals used for scientific or educational purposes; therefore, ethical approval was waived for this study. The care of the dogs was in accordance with the current standards. All owners signed a written informed consent.

All dogs underwent a complete staging work-up, as reported elsewhere (Marconato et al. 2014), and lymphadenectomy of an enlarged peripheral lymph node (LN). The histotype was confirmed by histologic examination and IHC (CD3 and CD20) (Valli et al. 2011).

The following clinico-pathologic data were recorded: signalment, clinical stage, substage, flow cytometric (FC) assessment of LN, level of peripheral blood (PB) and bone marrow (BM) infiltration, serum LDH activity, pre-treatment with steroids, treatment, and cause of death (Marconato et al. 2013; Marconato et al. 2015; Marconato et al. 2019; Martini et al. 2019; Giannuzzi et al. 2022). The extent of PB and BM infiltration was reported as the percentage of cells with the same scatter properties and antigen expression than those found in the LN out of total CD45+ events (all leukocytes) (Marconato et al. 2013). Dogs with at least 1% of PB and/or BM infiltration were arbitrarily classified as having stage V disease. *TP53* mutational status was assessed as previously described (Giannuzzi et al. 2022).

Depending on owner’s preference, dogs were treated either with chemotherapy, consisting of a CHOP-based protocol (including L-asparaginase, vincristine, cyclophosphamide, doxorubicin, lomustine and prednisone), or chemo-immunotherapy (CHOP-based protocol plus the autologous vaccine APAVAC^®^) (Marconato et al. 2014). Treatment response was evaluated at each therapeutic session, lasting for at least 28 days, and was classified as complete remission (CR), partial remission (PR), stable disease (SD) or progressive disease (PD) according to predefined criteria (Vail et al. 2010). For dogs obtaining CR, end-staging was carried out at the end of the protocol and every clinical, radiological, ultrasonographic or laboratory investigation that disclosed abnormalities at pre-treatment staging was repeated. After treatment, follow-up evaluation consisted of physical examination, including cytologic evaluation of peripheral LNs and imaging, if clinically indicated, on a monthly basis during the first year and every 2-3 months thereafter. Relapse was defined as the clinical reappearance and cytologic evidence of lymphoma with or without FC confirmation in any anatomical site in dogs having experienced CR. Time to progression (TTP) was measured as the interval between initiation of treatment and relapse, whereas lymphoma-specific survival (LSS) was calculated as the interval between initiation of treatment and lymphoma-related death (Vail et al. 2010).

### RNAscope^®^ ISH and immunohistochemistry

RNAscope^®^ ISH assay (Advanced Cell Diagnostics [ACD] Inc, Santa Monica, CA) was performed on the Ventana Discovery Ultra autostainer (Roche, Ventana Medical System, Inc., Tucson, AZ, USA) according to the manufacturer’s instruction. The RNAscope^®^ 2.5 VS probe Cl-TP53-C1, Canis lupus familiaris tumor protein p53 (*TP53*) mRNA (ACD), targeting the region 128 – 1197 of the canine *TP53* mRNA (Accession No: NM_001389218.1), was used. Briefly, 4-µm-thick FFPE sections were deparaffinized and pre-treated with heat and protease before hybridization. The probes were incubated at 42 °C for 2 h. The final deposit was detected as a red, pinpoint precipitate using the mRNA RED Detection Kit (Roche). For each sample, 2 further serial sections were stained using probes for Cl-PPIB [peptidylprolyl isomerase B (cyclophilin B)] and dapB (Bacillus subtilis dihydrodipicolinate reductase gene). PPIB was used as an endogenous control to evaluate RNA integrity, while the bacterial gene dapB served as a negative control to evaluate nonspecific background staining. The quantification of positivity was performed counting the number of positive dots per cell, applying the ACD semi-quantitative scoring system for RNAscope^®^, as follows:

- score 0: no signal or less than 1 dot every 10 cells;
- score 1: 1 to 3 dots per cell;
- score 2: 4 to 9 dots per cell;
- score 3: 10 to 15 dots per cell and/or a percentage <10% of dots are in clusters;
- score 4: more than 15 dots per cell and/or a percentage >10% of dots are in clusters.

IHC for p53 was performed on the Ventana Discovery Ultra autostainer (Roche) using a monoclonal antibody (clone PAb240; BD Pharmigen) previously validated in canine tissues (Brunetti et al. 2023). Briefly, 3-μm-thick FFPE tissue sections were deparaffinized in aqueous-based detergent solution (EZ Prep, Ventana), followed by antigen retrieval with Discovery CC1 (Roche) at 95 °C for 32 min. Slides were incubated with a 1:50 primary antibody solution for 40 min RT. Detection was performed with the Discovery OmniMap anti-Ms HRP (Roche) and Chromomap DAB (Roche). Sections were counterstained with Mayer’s hematoxylin. Tumor sections with omission of the primary antibodies were included in each run. Whole sections were evaluated for the detection of cells with positive p53 nuclear staining. Tumors were regarded as positive in the presence of at least one positive neoplastic cell, with either strong or faint staining. The percentage of p53 positive cells was calculated counting the number of positive cells in at least 1,000 cells. In tumors with variability in p53 staining in different fields, areas with a higher density of positive cells were counted (Fernandez-Pol et al. 2017).

### Statistical and survival analysis

All the analyses were conducted in the R environment (R Software v.4.1.2). The associations between the clinico-pathological features were investigated by means of Fisher’s exact test for categorical variables and Student’s t-test or Wilcoxon rank-sum test for continuous variables, depending on normality data distribution previously assessed by Shapiro-Wilk test. The diagnostic performance of IHC to detect *TP53* mutation, compared to sequencing approaches, was evaluated by calculating the sensitivity, specificity, accuracy and predictive value, and the diagnostic odds ratio. The correlation between RNAscope^®^ scores and the percentage of BM and PB infiltration was tested by Spearman rank correlation testing. Survival analysis was conducted using the univariable Cox proportional-hazards model (*survival* and *survminer* packages) in order to test the impact of the following clinico-pathological features on TTP and LSS: breed (pure vs mixed), sex (female vs male), age (median used as cut-off), weight (median used as cut-off), stage (III, IV, V), substage (a vs b), PB infiltration (%), BM infiltration (%), presence of BM infiltration (yes vs no), serum LDH activity (normal vs increased), pretreatment with steroids (yes vs no), *TP53* mutational status (mutated vs wild type), *TP53* mRNA expression, and p53 protein expression (neg vs pos). A cut-off of *p* < 0.01 was used to screen the variables for multivariable analysis. Median TTP and LSS were estimated using Kaplan-Meier method.

## Results

### Selection of dogs

Thirty-seven dogs were included in the study (Supplementary Table S1), comprising 7 (18.9%) mixed-breed dogs, 4 (10.8%) German shepherds, 4 (10.8%) Golden retrievers, 3 (8.1%) Rottweilers, and other pure breeds which were represented once or twice. Nineteen (51.4%) dogs were males and 18 (48.6%) were females. Median age was 7 years (range, 3-15), and median weight was 25 kg (range, 4.5-50.0).

All dogs had a diagnosis of cDLBCL: 2 (5.4%) had stage III disease, 12 (32.4%) stage IV, and 23 (62.2%) stage V. Twenty-six (70.3%) dogs showed no symptoms (substage a), whereas 11 (29.7%) had substage b disease. Ten (27.0%) dogs had received prednisone before undergoing disease staging. Regarding LDH, it was increased in 22 (59.5%) dogs. FC results for PB and BM were available for all cases. Overall, 20 (54.1%) dogs had PB infiltration, and 23 (62.2%) dogs had BM involvement. Four dogs with BM involvement had no circulating neoplastic cells, whereas 1 dog with PB involvement had no BM infiltration. Median PB infiltration at diagnosis was 3.6% (range, 1.0-55.5). Median BM infiltration at diagnosis was 3.2% (range, 1.2-50.0).

Regarding treatment, 26 (70.3%) dogs received chemo-immunotherapy and 11 (29.7%) chemotherapy. Twenty-seven (72.9%) dogs obtained CR, 7 (18.9%) PR, 1 (2.7%) dog was stable and 2 (5.4%) progressed. Median TTP was 98 days (range, 1-1403). At data analysis closure, all dogs had died, 34 (91.9%) for lymphoma-related causes and 3 (8.1%) for unrelated causes. Median LSS was 228 days (range, 22-1403).

Eleven (29.7%) dogs harbored 12 somatic *TP53* mutations within the DNA-binding domain, while 26 (70.3%) were wild-type. Among the mutated cases, 9 dogs exhibited missense mutations, one had both a missense mutation and frameshift deletion, and one harbored a frameshift insertion (Supplementary Table S2). All missense variants were predicted as deleterious by SIFT.

### TP53 mRNA and p53 protein expression

**Figure 1.**
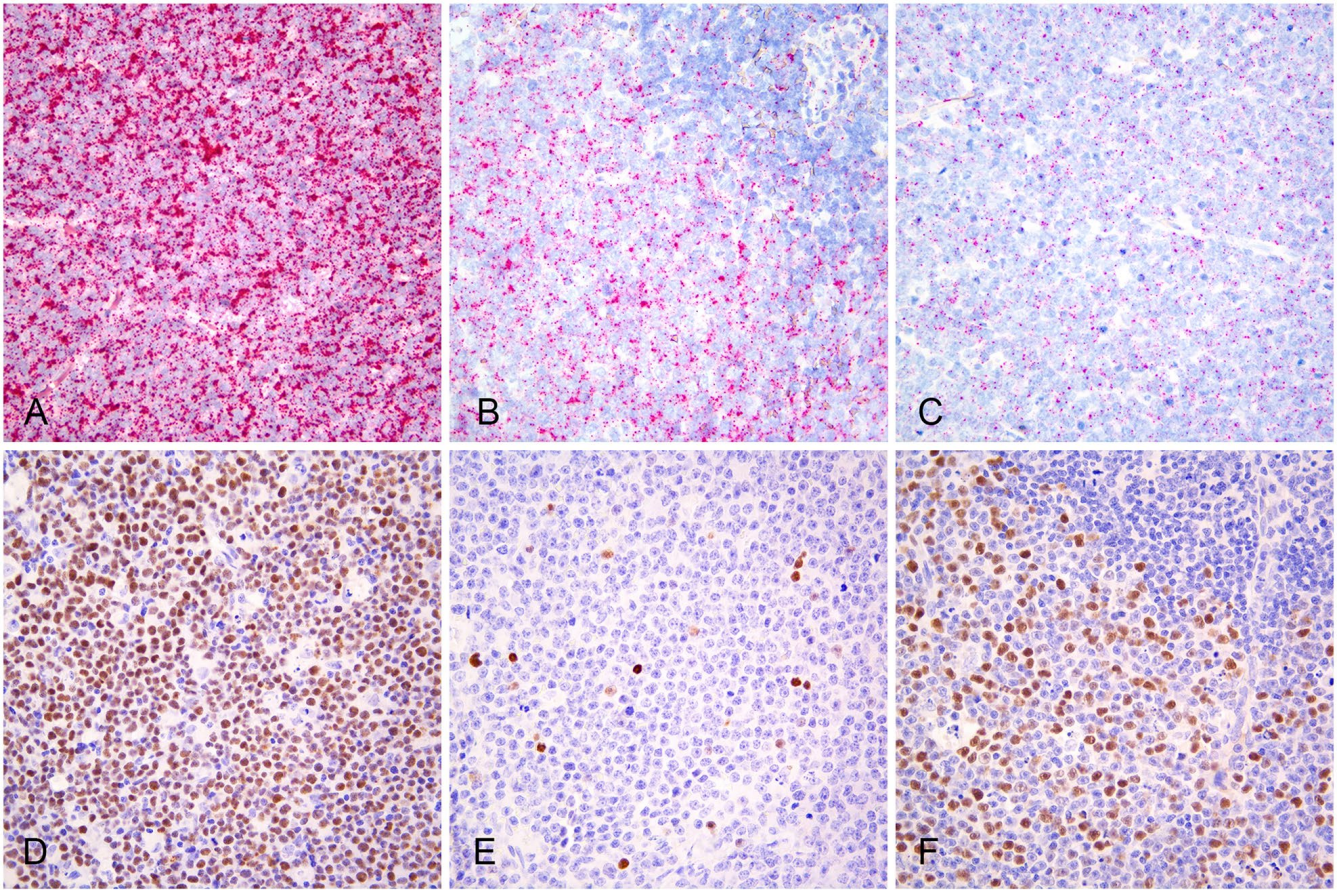
*TP53* RNAscope^®^ *In situ* hybridization and p53 immunohistochemistry in canine diffuse large B-cell lymphomas. (a) *TP53* RNAscope^®^ score 4. Case 23. (b) *TP53* RNAscope^®^ score 3 in neoplastic cells and residual small lymphocytes with lower *TP53* expression (upper right, Supplementary Figure S3). Case 1. (c) *TP53* RNAscope^®^ score 2. Case 2. (d) p53 strong nuclear immunohistochemical expression in the majority of neoplastic cells. Case 28. (e) Scattered neoplastic cells exhibiting p53 expression with variable staining intensity. Case 34. (f) Variable p53 expression in neoplastic cells and negative residual small lymphocytes (upper right, Supplementary Figure S3). Case 31.

*TP53* expression was detected by RNAscope^®^ ISH in all analyzed cDLBCLs (37/37) (Figure 1(a–c)). Neoplastic cells exhibited diffuse *TP53* expression, with 18/37 (48.6%) cases displaying a score of 4 (Figure 1(a)), 13/37 (35.1%) a score of 3 (Figure 1(b)), and 6/37 (16.2%) cases a score of 2 (Figure 1(c)). Additionally, *TP53* RNAscope^®^ ISH expression was detected in aggregates of small round cells with condensed nuclei, consistent with tumor infiltrating lymphocytes or residual normal lymphocytes, where the number of dots per cell was lower than in neoplastic cells (Figure 1(b), Supplementary Figure S3). Furthermore, low *TP53* expression was observed in endothelial cells, vascular smooth muscle cells, and spindle cells of connective tissue.

Ten out of 37 (27.0%) cDLBCLs tested positive for p53 by IHC. Heterogeneous staining intensity and positive cell density were observed both within different regions of the same tumor section and among different cases (Figure 1(d, f)). The percentage of tumor cells exhibiting positive p53 nuclear staining ranged from 0.3% to 84.8%. Specifically, among the 10 positive cases, 6 exhibited percentages below 5%, one had 8.5%, one had 47.8%, and two cases had percentages higher than 80% (Supplementary Table S1). Agglomerates of small lymphocytes or cells of the stroma and adjacent fibrous and adipose tissue consistently showed negative staining (Figure 1(f), Supplementary Figure S3).

### TP53 RNAscope^®^ ISH, p53 IHC and TP53 mutational status

**Figure 2.**
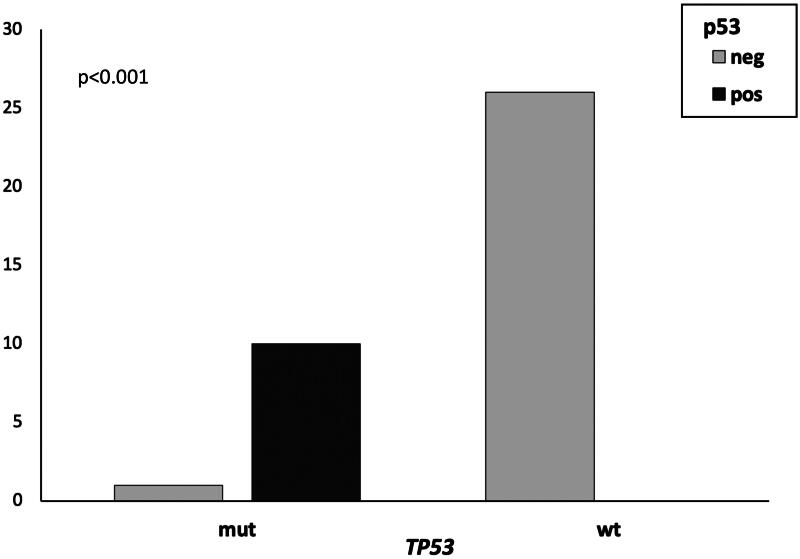
p53-positive vs p53-negative canine diffuse large B-cell lymphomas by immunohistochemistry, with mutated (mut) and wild-type (wt) *TP53*. All mutated cases, except for one, exhibited p53 protein expression, while all wild-type cases were negative (*p* < 0.001).

The expression of *TP53* detected by RNAscope^®^ ISH was not influenced by the mutational status. Conversely, a significant association was observed between *TP53* mutation and p53 expression by IHC (*p* < 0.001). Specifically, all mutated cases, except for one, exhibited p53 protein expression, while all wild-type cases were negative (Figure 2). Mutated cDLBCL cases with positive IHC harbored *TP53* missense mutations, whereas the only negative mutated case exhibited a frameshift insertion. The case with both missense mutation and frameshift deletion had 2.5% of p53 positive cells. The accuracy of p53 IHC in detecting *TP53* mutation (Table 1) was 97.3% (95% CI 85.84-99.93), with robust sensitivity, specificity, and diagnostic odds ratio of 371 (95% CI 13.9671-9854.6517; *p* = 0.0004). No significant association between RNAscope^®^ scores and immunohistochemical p53 expression was identified.

**Table 1.** Accuracy of cDLBCL *TP53* mutation status assignment based on p53 immunohistochemical testing, contingency table.

	*TP53* Mut	*TP53* WT	Total	
IHC p53 +	10 (TP)	0 (FP)	10	PPV 100% (69.15%–100%)
IHC p53 -	1 (FN)	26 (TN)	27	NPV 96.30% (80.05%–99.41%)
Total	11	26	37	
	Sensitivity 90.91% (58.72%–99.77%)	Specificity 100% (86.77%–100%)	Accuracy 97.30% (85.84%–99.93%)	

TP, true positive; FP, false positive; FN, false negative; TN, true negative; PPV, positive predictive value; NPV, negative predictive value. (95% Confidence Interval)

### TP53 RNAscope^®^ ISH, p53 IHC, TP53 mutational status and clinico-pathological features

We investigated the associations of *TP53* expression detected by RNAscope^®^ ISH and p53 IHC results with the clinico-pathological features of the dogs. The *TP53* RNAscope^®^ score was significantly higher in dogs diagnosed with stage V disease compared to those with stage III and IV (*p* = 0.004). Additionally, *TP53* RNAscope^®^ score positively correlated with the percentage of BM neoplastic infiltration (*p* = 0.016). Conversely, no significant associations were found between p53 immunohistochemical expression and the clinical features.

According to univariable Cox proportional-hazards analysis, both TTP and LSS were significantly shorter in dogs treated with chemotherapy alone compared to those treated with chemo-immunotherapy (median TTP: 34 vs 202 days, *p* = 0.007; median LSS: 60 vs 302 days, *p* = 0.003). Dogs bearing *TP53* mutations also exhibited significantly shorter TTP and LSS compared to those with wild-type *TP53* (median TTP: 53 vs 199 days, *p* = 0.004; median LSS: 69 vs 290 days, *p* = 0.001). Additionally, tumors expressing p53 had significantly shorter TTP and LSS compared to those not expressing the protein (median TTP: 52 vs 196 days, *p* = 0.002; median LSS: 66 vs 278 days, *p* = 0.002). Moreover, symptomatic dogs (substage b) showed significantly shorter TTP compared to dogs without symptoms (substage a) (median TTP: 69 vs 188 days, *p* = 0.047), while the presence of BM infiltration was associated with shorter LSS (median LSS: 74 vs 278 days, *p* = 0.04) (Supplementary Table S4).

Nevertheless, in multivariable analysis (Supplementary Table S4), only treatment emerged as a significant factor impacting both TTP (*p* = 0.002) and LSS (*p* < 0.001). Other factors, such as *TP53* mutations and p53 immunohistochemical expression, did not retain their significance in influencing TTP or LSS when considered alongside the treatment.

## Discussion

For decades, p53 has been regarded as the ‘Guardian of the Genome’ and over time it has remained one of the most extensively studied genes (Lane 1992). In humans, *TP53* mutations are found in approximately 50% of tumors (Vogelstein et al. 2000).

Even though fewer studies have been conducted compared to the human counterparts, *TP53* mutations have also been described for several canine tumors (Alsaihati et al. 2021; Das et al. 2023; Estabrooks et al. 2023). Approximately 16% of canine B-cell lymphomas (Fanelli A. et al. unpublished data) harbor *TP53* mutations at the time of diagnosis, with roughly one in four cases of cDLBCL exhibiting mutated *TP53* (Giannuzzi et al. 2022). Furthermore, the presence of *TP53* mutations in cDLBCL correlates with an unfavorable outcome (Giannuzzi et al. 2022), highlighting the importance of incorporating such genetic information to inform clinical management and therapeutic decisions.

In this study, we investigated the role of p53 in cDLBCL using a comprehensive approach that included *TP53* mutational analysis, RNAscope^®^ ISH and p53 IHC. Genetic testing of *TP53* by sequencing approaches served as the benchmark for comparison with IHC and RNAscope^®^ results.

In the study cohort, 30% of cDLBCL showed *TP53* aberrations, predominantly represented by missense variations affecting the DNA-binding domain. These mutations mirrored those observed in human DLBCL (Xu-Monette et al. 2012), emphasizing the evolutionary conservation of genomic alterations in lymphoid malignancies across species.

The agreement observed between p53 immunohistochemical expression and *TP53* mutations highlights the potential utility of IHC as a convenient and cost-effective tool for routine diagnostics to identify mutations in this gene. IHC for p53 protein exhibited high accuracy, specificity, and sensitivity in detecting *TP53* mutations. This is particularly noteworthy given the challenges and limitations associated with the routine use of DNA sequencing in veterinary practice.

Biologically, p53 immunohistochemical staining indicates that *TP53* mutations in cDLBCL lead to the accumulation of mutant p53 protein. The majority of *TP53*-mutated cDLBCL harbored missense mutations, potentially resulting in the production of stabilized mutant p53, as in human tumors. These mutations commonly confer GOF properties, which play crucial roles in promoting invasion, metastasis, and inducing structural chromosomal changes, ultimately leading to high levels of genomic instability (Zhang et al. 2020). The molecular mechanisms driving the oncogenic functions of mutant p53 proteins involve their interaction with other transcription factors and an increased ability to bind to other p53 family members, such as p63 and p73 (Gaiddon et al. 2001). Recent advancements in therapeutic strategies targeting p53 GOF have been achieved and should be considered in dogs as well (Zhang et al. 2020). These include the elimination of mutant p53, restoration or reactivation of wild-type p53, destabilization of mutant p53, or inhibition of downstream signaling resulting from mutant p53 GOF, thereby inducing synthetic lethality in cells expressing mutant p53 (Zhang et al. 2020).

In our study, p53 immunohistochemical positivity consistently correlated with *TP53* mutation, regardless of the variable percentage of p53-positive cells. In human tumors, both p53 overexpression and complete absence (null pattern) are considered aberrant and strongly correlated with the presence of a mutation (Nenutil et al. 2005; Köbel et al. 2019). A third aberrant cytoplasmic pattern has been recently identified in human gynecologic epithelial malignancies (Köbel et al. 2016). While p53 overexpression by IHC is commonly linked to GOF activities, p53 null or cytoplasmic patterns have been associated with mutations inducing p53 LOF (i.e. nonsense, frameshift insertions or deletions) (Köbel et al. 2016). Conversely, low to intermediate p53 expression is considered a normal/wild-type pattern (Köbel et al. 2019). Several stimuli such as hypoxia, oxidative stress, or chemotherapeutic agents can indeed stabilize wild-type p53, leading to positive staining in the absence of mutation (Suh et al. 2011). Commonly used cut-off values for identifying p53 overexpression vs wild-type pattern are 5% or 10% of positively stained cells, which are also utilized for canine tumors (Yemelyanova et al. 2011; Dhaliwal et al. 2013; Russell et al. 2018; Brunetti et al. 2023), although their application is controversial as the interpretation of p53 IHC varies greatly among different tumor types, p53 antibodies, and protocols (Park et al. 2022). In this study, cDLBCL cases with percentages of positive cells below 5% or 10% also harbored *TP53* mutation, contrary to previous assumptions (Sokołowska et al. 2005). This observation might suggest that in a subset of cases, p53 missense mutation results in a modest accumulation of the mutant protein. Moreover, all cDLBCL with wild-type *TP53* displayed a null pattern by IHC, and complete absence of p53 expression was also observed in non-tumor cells, such as normal stromal fibroblasts, endothelial cells, and small lymphocytes, as previously observed in canine tissues (Albaric et al. 2001). Contrarily, in human cancer, occasional p53 positivity in non-tumor cells may serve as internal control (Köbel et al. 2019). These findings may result from several factors, including the IHC protocol, and diverse p53 metabolism.

In our study, p53 IHC was unable to distinguish a cDLBCL harboring *TP53* frameshift insertion from cases with wild-type *TP53,* all exhibiting negative p53 immunohistochemical staining. This finding is in agreement with observations in human tumors where these mutations induce absent or reduced p53 protein production with LOF, and are associated to a null IHC pattern (Köbel et al. 2016). Contrarily, the cDLBCL case harboring both missense mutation and frameshift deletion included in this study, exhibited low percentages of p53 positive cells (<5%), similar to other cases with only missense mutations. This suggests that the combined mutation induced similar accumulation of mutant p53. However, further investigations are warranted to explore the correlation between p53 immunohistochemical expression and different mutation types in larger cohorts of cDLBCL encompassing different *TP53* variants.

In this study we also assessed the feasibility of measuring mRNA p53 through RNAscope^®^ ISH and to compare the resulting scores with the mutational status, protein expression, and clinical characteristics of the dogs. The detection of *TP53* mRNA in all analyzed cases suggests a widespread expression of this gene in neoplastic cells, even with a moderate heterogeneity across different cases. Notably, the majority of cases exhibited scores of 3 and 4, indicative of robust *TP53* expression. The lower *TP53* RNAscope^®^ expression identified in aggregates of small lymphocytes or non-neoplastic cells, such as endothelial cells, vascular smooth muscle cells, and spindle cells of fibroconnective tissue, may reflect the normal p53 gene expression, possibly heightened by microenvironmental stressors.

Despite the heterogeneity in *TP53* transcript expression, *TP53* mutational status did not influence the expression detected by RNAscope^®^. This intriguing finding raises questions about the regulatory mechanisms governing *TP53* expression in cDLBCL and warrants further investigation into the factors influencing its expression at the RNA level. Moreover, no correlations between *TP53* RNAscope^®^ scores and p53 protein were identified. This implies a potential divergence in the regulation of *TP53* at the RNA and protein levels, indicating the need for a comprehensive understanding of the transcriptional and post-transcriptional mechanisms governing *TP53* expression (Freeman and Espinosa 2013).

In this study we also assessed whether *TP53* mRNA and p53 protein expression could serve as prognostic markers. A significant association between *TP53* RNAscope^®^ scores and the disease stage was identified. Specifically, dogs diagnosed with stage V disease displayed higher *TP53* RNAscope^®^ scores in comparison to those with stage III and IV disease. However, this finding has limited practical relevance in the clinical setting. Given the availability of established diagnostic tools, such as flow cytometry, capable of determining PB and BM infiltrations (Marconato et al. 2013; Marconato et al. 2015), the incremental value of incorporating *TP53* RNAscope^®^ scores into the clinical work-up of dogs affected by DLBCL appears modest. No correlation was observed between *TP53* mRNA expression and outcome data.

p53 immunohistochemical expression was associated with poor outcome in univariable analysis, similarly to *TP53* mutational status. However, these correlations were not confirmed in the multivariable analysis. Associations among *TP53* mutation and poor clinical outcome has been previously reported both in humans and dogs (Giannuzzi et al. 2022; Landsburg et al. 2023). Moreover, a previous study has reported a correlation between p53 immunohistochemical expression and decreased overall survival in a cohort of canine lymphomas including cDLBCL (Dhaliwal et al. 2013). Several factors, such as the small sample size, the high variability in patient characteristics, treatment regimens (chemotherapy vs chemo-immunotherapy), and follow-up protocols, could have contributed to the lack of correlation observed between *TP53* mutational status and p53 IHC with outcome data in our study. Furthermore, *TP53* mutations often occur in conjunction with additional mutations or alterations in key signaling pathways that might influence tumor behavior and treatment response.

In conclusion, our findings indicate that the p53 IHC assay is a reliable and cost-effective surrogate for identifying *TP53* mutations in cDLBCL. Conversely, the ISH approach may be effective to investigate the regulation of *TP53* expression at a morphological level. Further investigation in larger cDLBCL cohorts is warranted to deepen the transcriptional and post-transcriptional mechanisms governing *TP53* expression, as well as to assess the potential prognostic significance and clinical utility of p53 IHC in guiding treatment decisions.

## Supplementary Material

Supplemental Material

## References

[CIT0001] Albaric O, Bret L, Amardeihl M, Delverdier M. 2001. Immunohistochemical expression of p53 in animal tumors: a methodological study using four anti-human p53 antibodies. Histol Histopathol. 16(1):113–121. doi: 10.14670/HH-16.113. 11193185

[CIT0002] Alsaihati BA, Ho KL, Watson J, Feng Y, Wang T, Dobbin KK, Zhao S. 2021. Canine tumor mutational burden is correlated with TP53 mutation across tumor types and breeds. Nat Commun. 12(1):4670. doi: 10.1038/s41467-021-24836-9. 34344882 PMC8333103

[CIT0003] Aresu L. 2016. Canine lymphoma, more than a morphological diagnosis: what we have learned about diffuse large B-cell lymphoma. Front Vet Sci. 3:77. doi: 10.3389/FVETS.2016.00077. 27630997 PMC5006005

[CIT0004] Aresu L, Ferraresso S, Marconato L, Cascione L, Napoli S, Gaudio E, Kwee I, Tarantelli C, Testa A, Maniaci C, et al. 2019. New molecular and therapeutic insights into canine diffuse large B-cell lymphoma elucidates the role of the dog as a model for human disease. Haematologica. 104(6):e256–e259. doi: 10.3324/haematol.2018.207027. 30545928 PMC6545862

[CIT0005] Attardi LD, Boutelle AM. 2024. Targeting p53 gain-of-function activity in cancer therapy: a cautionary tale. Cell Death Differ. 31(2):133–135. doi: 10.1038/s41418-023-01253-7. 38151545 PMC10850540

[CIT0006] Avery AC. 2020. The genetic and molecular basis for canine models of human leukemia and lymphoma. Front Oncol. 10:23. doi: 10.3389/fonc.2020.00023. 32038991 PMC6992561

[CIT0007] Bártek J, Bártková J, Vojtĕsek B, Stasková Z, Lukás J, Rejthar A, Kovarík J, Midgley CA, Gannon JV, Lane DP. 1991. Aberrant expression of the p53 oncoprotein is a common feature of a wide spectrum of human malignancies. Oncogene. 6(9):1699–1703. 1923535

[CIT0008] Brunetti B, de Biase D, Dellapina G, Muscatello LV, Ingravalle F, Tura G, Bacci B. 2023. Validation of p53 immunohistochemistry (PAb240 clone) in canine tumors with next-generation sequencing (NGS) analysis. Animals (Basel). 13(5):899. doi: 10.3390/ani13050899. 36899756 PMC10000222

[CIT0009] Childress MO, Ramos-Vara JA, Ruple A. 2018. Retrospective analysis of factors affecting clinical outcome following CHOP-based chemotherapy in dogs with primary nodal diffuse large B-cell lymphoma. Vet Comp Oncol. 16(1):E159–E168. doi: 10.1111/VCO.12364. 29152834

[CIT0010] Das S, Idate R, Lana SE, Regan DP, Duval DL. 2023. Integrated analysis of canine soft tissue sarcomas identifies recurrent mutations in TP53, KMT genes and PDGFB fusions. Sci Rep. 13(1):10422. doi: 10.1038/S41598-023-37266-Y. 37369741 PMC10300023

[CIT0011] Dhaliwal RS, Kitchell BE, Ehrhart EJ, Valli VE, Dervisis NG. 2013. Clinicopathologic significance of histologic grade, Pgp, and p53 expression in canine lymphoma. J Am Anim Hosp Assoc. 49(3):175–184. doi: 10.5326/JAAHA-MS-5843. 23535752

[CIT0012] Estabrooks T, Gurinovich A, Pietruska J, Lewis B, Harvey G, Post G, Lambert L, Miller A, Rodrigues L, White ME, et al. 2023. Identification of genomic alterations with clinical impact in canine splenic hemangiosarcoma. Vet Comp Oncol. 21(4):623–633. doi: 10.1111/VCO.12925. 37734854

[CIT0013] Fanelli A, Marconato L, Licenziato L, Minoli L, Rouquet N, Aresu L. 2022. POT1 mutations are frequent and associated with Ki-67 index in canine diffuse large B-cell lymphoma. Front Vet Sci. 9:968807. doi: 10.3389/FVETS.2022.968807. 36016811 PMC9396242

[CIT0014] Fernandez-Pol S, Ma L, Ohgami RS, Arber DA. 2017. Immunohistochemistry for p53 is a useful tool to identify cases of acute myeloid leukemia with myelodysplasia-related changes that are TP53 mutated, have complex karyotype, and have poor prognosis. Mod Pathol. 30(3):382–392. doi: 10.1038/MODPATHOL.2016.206. 27934876

[CIT0015] Freeman JA, Espinosa JM. 2013. The impact of post-transcriptional regulation in the p53 network. Brief Funct Genomics. 12(1):46–57. doi: 10.1093/BFGP/ELS058. 23242178 PMC3548162

[CIT7960076] Gaiddon C, Lokshin M, Ahn J, Zhang T, Prives C. 2001. A subset of tumor-derived mutant forms of p53 down-regulate p63 and p73 through a direct interaction with the p53 core domain. Mol Cell Biol. 21(5):1874–1887. doi: 10.1128/MCB.21.5.1874-1887.2001. 11238924 PMC86759

[CIT0016] Gamblin RM, Sagartz JE, Couto CG. 1997. Overexpression of p53 tumor suppressor protein in spontaneously arising neoplasms of dogs. Am J Vet Res. 58(8):857–863. doi: 10.2460/ajvr.1997.58.08.857. 9256970

[CIT0017] Giannuzzi D, Marconato L, Fanelli A, Licenziato L, De Maria R, Rinaldi A, Rotta L, Rouquet N, Birolo G, Fariselli P, et al. 2022. The genomic landscape of canine diffuse large B-cell lymphoma identifies distinct subtypes with clinical and therapeutic implications. Lab Anim (NY). 51(7):191–202. doi: 10.1038/s41684-022-00998-x. 35726023

[CIT0018] Keller SM, Schade B, Rickenbacher AB, Brugnera E, Wergin MC, Müller EJ, Suter MM, Guscetti F. 2007. A comprehensive test system to identify suitable antibodies against p53 for immunohistochemical analysis of canine tissues. J Comp Pathol. 137(1):59–70. doi: 10.1016/j.jcpa.2007.04.021. 17629968

[CIT0019] Köbel M, Piskorz AM, Lee S, Lui S, LePage C, Marass F, Rosenfeld N, Masson AMM, Brenton JD. 2016. Optimized p53 immunohistochemistry is an accurate predictor of TP53 mutation in ovarian carcinoma. J Pathol Clin Res. 2(4):247–258. doi: 10.1002/CJP2.53. 27840695 PMC5091634

[CIT0020] Köbel M, Ronnett BM, Singh N, Soslow RA, Gilks CB, McCluggage WG. 2019. Interpretation of p53 immunohistochemistry in endometrial carcinomas: toward increased reproducibility. Int J Gynecol Pathol. 38(Suppl. 1):S123–S131. doi: 10.1097/PGP.0000000000000488. 29517499 PMC6127005

[CIT0021] Landsburg DJ, Morrissette JJD, Nasta SD, Barta SK, Schuster SJ, Svoboda J, Chong EA, Bagg A. 2023. TP53 mutations predict for poor outcomes in patients with newly diagnosed aggressive B-cell lymphomas in the current era. Blood Adv. 7(23):7243–7253. doi: 10.1182/bloodadvances.2023011384. 37851898 PMC10698538

[CIT0022] Lane DP. 1992. p53, guardian of the genome. Nature. 358(6381):15–16. doi: 10.1038/358015a0. 1614522

[CIT0023] Levine AJ. 2019. The many faces of p53: something for everyone. J Mol Cell Biol. 11(7):524–530. doi: 10.1093/jmcb/mjz026. 30925588 PMC6736316

[CIT0024] Marconato L, Aresu L, Stefanello D, Comazzi S, Martini V, Ferrari R, Riondato F, Rouquet N, Frayssinet P, Sabattini S. 2019. Opportunities and challenges of active immunotherapy in dogs with B-cell lymphoma: a 5-year experience in two veterinary oncology centers. J Immunother Cancer. 7(1):146. doi: 10.1186/S40425-019-0624-Y. 31174615 PMC6554898

[CIT0025] Marconato L, Frayssinet P, Rouquet N, Comazzi S, Leone VF, Laganga P, Rossi F, Vignoli M, Pezzoli L, Aresu L. 2014. Randomized, placebo-controlled, double-blinded chemoimmunotherapy clinical trial in a pet dog model of diffuse large B-cell lymphoma. Clin Cancer Res. 20(3):668–677. doi: 10.1158/1078-0432.CCR-13-2283. 24300788

[CIT0026] Marconato L, Martini V, Aresu L, Sampaolo M, Valentini F, Rinaldi V, Comazzi S. 2013. Assessment of bone marrow infiltration diagnosed by flow cytometry in canine large B cell lymphoma: prognostic significance and proposal of a cut-off value. Vet J. 197(3):776–781. doi: 10.1016/J.TVJL.2013.05.003. 23735731

[CIT0027] Marconato L, Martini V, Stefanello D, Moretti P, Ferrari R, Comazzi S, Laganga P, Riondato F, Aresu L. 2015. Peripheral blood lymphocyte/monocyte ratio as a useful prognostic factor in dogs with diffuse large B-cell lymphoma receiving chemoimmunotherapy. Vet J. 206(2):226–230. doi: 10.1016/J.TVJL.2015.07.009. 26403958

[CIT0028] Martini V, Aresu L, Riondato F, Marconato L, Cozzi M, Stefanello D, Comazzi S. 2019. Prognostic role of non-neoplastic lymphocytes in lymph node aspirates from dogs with diffuse large B-cell lymphoma treated with chemo-immunotherapy. Res Vet Sci. 125:130–135. doi: 10.1016/J.RVSC.2019.06.003. 31212200

[CIT0029] Miller M, Shirole N, Tian R, Pal D, Sordella R. 2016. The evolution of TP53 mutations: from loss-of-function to separation-of-function mutants. J Cancer Biol Res. 4(4):1091. PMC5298884 28191499

[CIT0030] Nenutil R, Smardova J, Pavlova S, Hanzelkova Z, Muller P, Fabian P, Hrstka R, Janotova P, Radina M, Lane DP, et al. 2005. Discriminating functional and non-functional p53 in human tumours by p53 and MDM2 immunohistochemistry. J Pathol. 207(3):251–259. doi: 10.1002/PATH.1838. 16161005

[CIT0031] Park E, Han H, Choi SE, Park H, Woo HY, Jang M, Shim HS, Hwang S, Kang H, Cho NH. 2022. p53 immunohistochemistry and mutation types mismatching in high-grade serous ovarian cancer. Diagnostics (Basel). 12(3):579. doi: 10.3390/DIAGNOSTICS12030579. PMC8947437 35328131

[CIT0032] Russell DS, Jaworski L, Kisseberth WC. 2018. Immunohistochemical detection of p53, PTEN, Rb, and p16 in canine osteosarcoma using tissue microarray. J Vet Diagn Invest. 30(4):504–509. doi: 10.1177/1040638718770239. 29629647 PMC6505918

[CIT0033] Sokołowska J, Cywińska A, Malicka E. 2005. p53 expression in canine lymphoma. J Vet Med A Physiol Pathol Clin Med. 52(4):172–175. doi: 10.1111/J.1439-0442.2005.00707.X. 15882401

[CIT0034] Sueiro FAR, Alessi AC, Vassallo J. 2004. Canine lymphomas: a morphological and immunohistochemical study of 55 cases, with observations on p53 immunoexpression. J Comp Pathol. 131(2-3):207–213. doi: 10.1016/j.jcpa.2004.04.002. 15276860

[CIT0035] Suh YA, Post SM, Elizondo-Fraire AC, Maccio DR, Jackson JG, El-Naggar AK, Van Pelt C, Terzian T, Lozano G. 2011. Multiple stress signals activate mutant p53 in vivo. Cancer Res. 71(23):7168–7175. doi: 10.1158/0008-5472.CAN-11-0459. 21983037 PMC3320147

[CIT0036] Vail DM, Michels GM, Khanna C, Selting KA, London CA. 2010. Response evaluation criteria for peripheral nodal lymphoma in dogs (v1.0)–a Veterinary Cooperative Oncology Group (VCOG) consensus document. Vet Comp Oncol. 8(1):28–37. doi: 10.1111/J.1476-5829.2009.00200.X. 20230579

[CIT0037] Valli VE, Myint M, Barthel A, Bienzle D, Caswell J, Colbatzky F, Durham A, Ehrhart EJ, Johnson Y, Jones C, et al. 2011. Classification of canine malignant lymphomas according to the world health organization criteria. Vet Pathol. 48(1):198–211. doi: 10.1177/0300985810379428. 20861499

[CIT0038] Vogelstein B, Lane D, Levine AJ. 2000. Surfing the p53 network. Nature. 408(6810):307–310. doi: 10.1038/35042675. 11099028

[CIT0039] Wang F, Flanagan J, Su N, Wang LC, Bui S, Nielson A, Wu X, Vo HT, Ma XJ, Luo Y. 2012. RNAscope: a novel in situ RNA analysis platform for formalin-fixed, paraffin-embedded tissues. J Mol Diagn. 14(1):22–29. doi: 10.1016/J.JMOLDX.2011.08.002. 22166544 PMC3338343

[CIT0040] Xu-Monette ZY, Wu L, Visco C, Tai YC, Tzankov A, Liu WM, Montes-Moreno S, Dybkær K, Chiu A, Orazi A, et al. 2012. Mutational profile and prognostic significance of TP53 in diffuse large B-cell lymphoma patients treated with R-CHOP: report from an International DLBCL Rituximab-CHOP Consortium Program Study. Blood. 120(19):3986–3996. doi: 10.1182/BLOOD-2012-05-433334. 22955915 PMC3496956

[CIT0041] Xu J, Wang J, Hu Y, Qian J, Xu B, Chen H, Zou W, Fang JY. 2014. Unequal prognostic potentials of p53 gain-of-function mutations in human cancers associate with drug-metabolizing activity. Cell Death Dis. 5(3):e1108–e1108. doi: 10.1038/cddis.2014.75. 24603336 PMC3973211

[CIT0042] Yemelyanova A, Vang R, Kshirsagar M, Lu D, Marks MA, Shih IM, Kurman RJ. 2011. Immunohistochemical staining patterns of p53 can serve as a surrogate marker for TP53 mutations in ovarian carcinoma: an immunohistochemical and nucleotide sequencing analysis. Mod Pathol. 24(9):1248–1253. doi: 10.1038/MODPATHOL.2011.85. 21552211

[CIT0043] Zhang C, Liu J, Xu D, Zhang T, Hu W, Feng Z. 2020. Gain-of-function mutant p53 in cancer progression and therapy. J Mol Cell Biol. 12(9):674–687. doi: 10.1093/JMCB/MJAA040. 32722796 PMC7749743

[CIT0044] Zhou X, Santos GS, Zhan Y, Oliveira MMS, Rezaei S, Singh M, Peuget S, Westerberg LS, Johnsen JI, Selivanova G. 2022. Mutant p53 gain of function mediates cancer immune escape that is counteracted by APR-246. Br J Cancer. 127(11):2060–2071. doi: 10.1038/s41416-022-01971-8. 36138076 PMC9681866

