# Supplementary material for: Predictive value of TP53 RNAscope®in situ hybridization and p53 immunohistochemistry for TP53 mutational status in canine diffuse large B-cell lymphoma
Source: Vet Q. 2024 Sep 16;44(1):1–9. doi: 10.1080/01652176.2024.2403453 (PMC11407423; doi:10.1080/01652176.2024.2403453)
Supplement: Supplemental Material [file TVEQ_A_2403453_SM4870.zip › suppl_data/Supp_Captions.docx]

**Supplemental online material**

**Supplementary Table S1.** Clinico-pathological data of the 37 dogs with DLBCL included in the study.

**Supplementary Table S2**. *TP53* mutations identified in the cohort of 37 cDLBCL.

**Supplementary Figure S3.** *TP53* RNAscope^®^ *in situ* hybridization and p53 immunohistochemistry in residual normal lymphocytes within lymph nodes affected by canine diffuse large B-cell lymphoma. A. High-magnification image of Fig. 1B depicting lower *TP53* RNAscope^®^ expression in residual small lymphocytes (outlined by a dashed line) compared to neoplastic large lymphoid cells (*TP53* RNAscope^®^ score 3). Case 1. B. High-magnification image of Fig. 1F depicting p53-negative residual small lymphocytes (outlined by a dashed line) and variably p53-positive neoplastic large lymphoid cells by immunohistochemistry. Case 31.

**Supplementary Table S4.** Summary of survival analysis by univariable and multivariable Cox proportional-hazards.
