# Supplementary figures and images for: Predictive value of TP53 RNAscope®in situ hybridization and p53 immunohistochemistry for TP53 mutational status in canine diffuse large B-cell lymphoma
Source: Vet Q. 2024 Sep 16;44(1):1–9. doi: 10.1080/01652176.2024.2403453 (PMC11407423; doi:10.1080/01652176.2024.2403453)

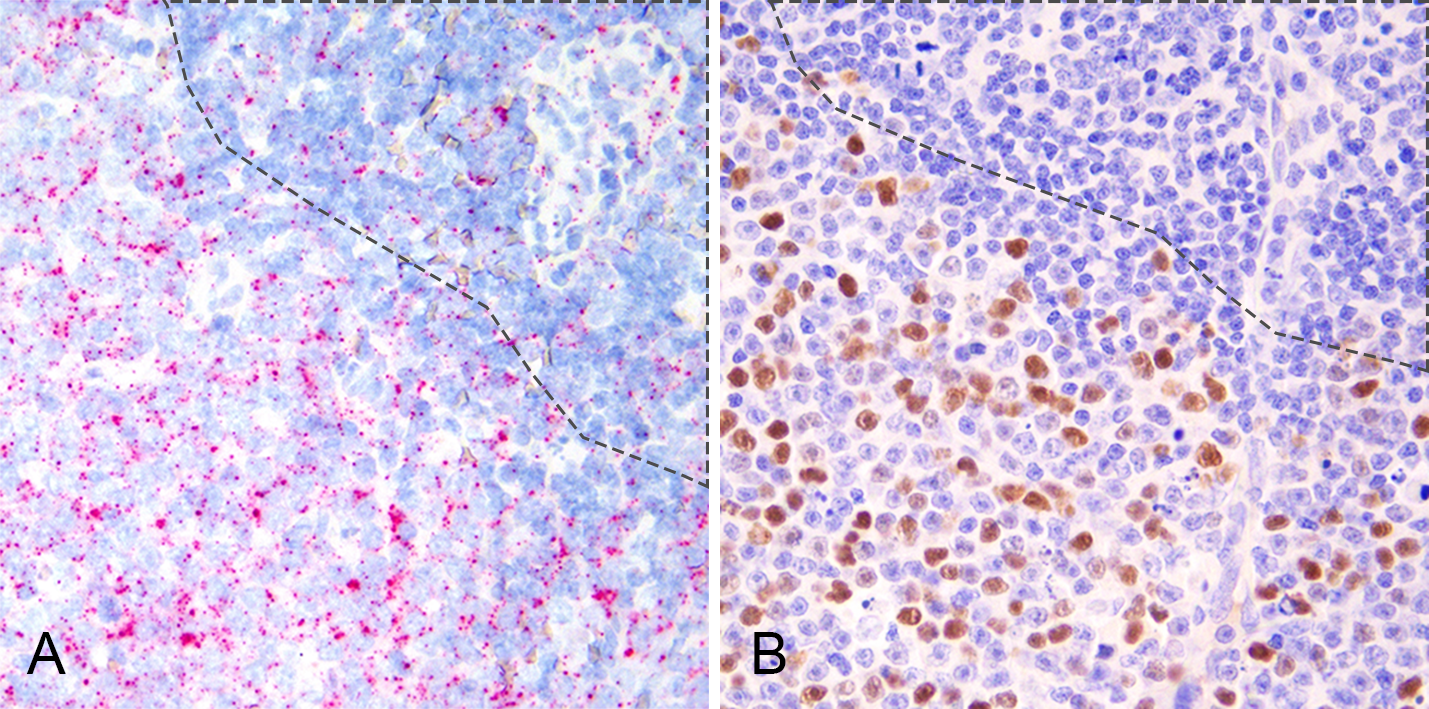

Supplement: Supplemental Material [file TVEQ_A_2403453_SM4870.zip › suppl_data/Supplemental Figure S3.tif]
